# Effective antitumor activity of 5T4‐specific CAR‐T cells against ovarian cancer cells in vitro and xenotransplanted tumors in vivo

**DOI:** 10.1002/mco2.34

**Published:** 2020-10-22

**Authors:** Cuiyu Guo, E Dong, Qinhuai Lai, Shijie Zhou, Guangbing Zhang, Mengdan Wu, Xiaozhu Yue, Yiran Tao, Yujia Peng, Jamel Ali, Ying Lu, Yuyin Fu, Weirong Lai, Zhixiong Zhang, Fanxin Ma, Yuqin Yao, Lantu Gou, Hanshuo Yang, Jinliang Yang

**Affiliations:** ^1^ State Key Laboratory of Biotherapy and Cancer Center/Collaborative Innovation Center for Biotherapy West China Hospital Sichuan University Chengdu Sichuan People's Republic of China; ^2^ West China‐California Research Center for Predictive Intervention Medicine West China Hospital Sichuan University Chengdu Sichuan People's Republic of China; ^3^ Healthy Food Evaluation Research Center/Sichuan University West China School of Public Health and West China Fourth Hospital Chengdu People's Republic of China; ^4^ Department of Chemical and Biomedical Engineering FAMU‐FSU College of Engineering Tallahassee Florida

**Keywords:** 5T4, CAR‐T cell immunotherapy, chimeric antigen receptor, ovarian cancer

## Abstract

Ovarian cancer is considered to be the most lethal gynecologic malignancy, and despite the development of conventional therapies and new therapeutic approaches, the patient's survival time remains short because of tumor recurrence and metastasis. Therefore, effective methods to control tumor progression are urgently needed. The oncofetal tumor‐associated antigen 5T4 (trophoblast glycoprotein, TPBG) represents an appealing target for adoptive T‐cell immunotherapy as it is highly expressed on the surface of various tumor cells, has very limited expression in normal tissues, and spreads widely in malignant tumors throughout their development. In this study, we generated second‐generation human chimeric antigen receptor (CAR) T cells with redirected specificity to 5T4 (5T4 CAR‐T) and demonstrated that these CAR‐T cells can elicit lytic cytotoxicity in targeted tumor cells, in addition to the secretion of cytotoxic cytokines, including IFN‐γ, IL‐2, and GM‐CSF. Furthermore, adoptive transfer of 5T4 CAR‐T cells significantly delayed tumor formation in xenografts of peritoneal and subcutaneous animal models. These results demonstrate the potential efficacy and feasibility of 5T4 CAR‐T cell immunotherapy and provide a theoretical basis for the clinical study of future immunotherapies targeting 5T4 for ovarian cancer.

## Graphical abstract

We generated a second‐generation CAR‐T cell targeting 5T4 with the costimulatory molecule of 4‐1BB. 5T4 CAR‐T cells had specific cytotoxicity against ovarian cancer cells and secreted cytotoxic cytokines, including IFN‐γ, IL‐2 and GM‐CSF in vitro. 5T4 CAR‐T cells could eradicate peritoneal and subcutaneous tumor models in vivo completely in a short time without recurrence.

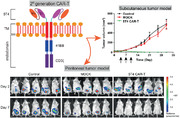

## 1 INTRODUCTION

Ovarian cancer is one of common gynecology cancers behind breast cancer and cervix uteri cancer^1^ and is the most fatal of gynecologic tumors. In 2018, there were a reported 295 414 new cases of ovarian cancer worldwide, including 184 799 deaths.^2^ At present, the common clinical treatments are surgery and postoperative platinum‐ and paclitaxel‐based chemotherapies. However, as this disease is usually discovered at an advanced stage and easily develops resistance to chemotherapy, the prognosis is poor, with a 5‐year survival rate below 35%. Therefore, new methods to control tumor progression are urgently needed. Bevacizumab is an antiangiogenic drug approved by the Food and Drug Administration (FDA) for platinum‐sensitive ovarian cancer in combination with chemotherapy, which has failed to improve overall survival rate in first‐line maintenance therapy.^3^ Recent scientific evidence has demonstrated that ovarian cancer is an immunogenic tumor that is under immune surveillance by the host immune system.^4^ Moreover, poly ADP‐ribose polymerase inhibitor Olaparib and programmed cell death protein ligand 1 (PD‐L1) antibody durvalumab were combined in the treatment of platinum‐sensitive recurrent ovarian cancer with BRCA mutations in a phase II clinical trial, and the overall response rate was 72%.^5^ These data suggest that immunotherapy may be a new method has good prospects for the treatment of ovarian cancer.

Immunotherapy has a revolutionary impact on the treatment of multiple malignancies and produced durable responses in most patients. Immunocheckpoint inhibitors and adoptive cell immunetherapy (ACI), in particular, have achieved impressive results. Up to the end of 2020, six monoclonal antibodies targeting programmed cell death protein 1 (PD‐1)/PD‐L1 and cytotoxic T‐lymphocyte antigen‐4 (CTLA‐4) have been approved by the FDA. Anti‐PD‐1/PD‐L1 monoclonal antibodies have demonstrated notable clinical efficacy because of their role in protecting against the exhaustion of CD8^+^ T cells.^6^ However, one limiting factor for these targeting antibodies is the lack of adequate effector T cells that can specifically recognize tumor antigens in cancer patients due to tumor cells downregulate the immune response function. The development of ACI as a cancer therapy began nearly 30 years ago to activate and amplify tumor‐infiltrating lymphocytes through tumor‐associated antigens (TAAs) in vitro.^7^ Nevertheless, existing TAAs are mostly endogenous and have low immunogenicity as they can adopt a variety of mechanisms to render immunologic tolerance.^8^

To solve this problem, T cells are artificially modified to express a chimeric antigen receptor (CAR), which consists of an extracellular antigen‐binding region, usually as a single‐chain variable fragment (scFv) derived from a monoclonal antibody (mAb), linked to an intracellular signaling domain. CAR‐T cells are able to directly trigger an immune response in a nonmajor histocompatibility complex‐restricted manner.^9^ First generation CAR‐T cells only contained a CD3ζ intracellular activation domain, which had limited amplification capacity and short survival times, resulting in insufficient IL‐2 production in vivo.^10^ Second‐generation CARs integrated costimulatory signaling domains, such as CD28, 4‐1BB (CD137), or OX40 (CD134),^11^, ^12^, ^13^ thus cytotoxicity and persistence of T cells were improved. In 2017, the FDA approved two CD19‐targeted CAR‐T cells: Novartis’ Kymriah and Kate's Yescarta for treating B‐cell lymphoma,^14^, ^15^ which brought CAR‐T cell immunotherapy to the forefront of standard cancer treatment. Unfortunately, in contrast to the inspirational progress in treating hematologic malignancies with CAR‐T cells, their curative effect on solid tumors has not been significant thus far. Perhaps due to the hostile tumor microenvironment, CAR‐T cells become exhausted before migrating and infiltrating into the tumor tissues.^16^ In addition, solid tumors tend to be heterogeneous, making it more difficult for single‐target CAR‐T cells to completely eradicate cancer cells.^17^

Improving the efficacy of CAR‐T cells in solid tumors and reducing their on‐target/off‐target toxicity is a critical challenge limiting the widespread application of CAR‐T cell therapy. To overcome these issues, selection of appropriate tumor‐targeting antigens is the key step in designing CARs. 5T4 is a highly *N*‐glycosylated transmembrane protein with a molecular weight of 72 kD, whose gene is found on chromosome 6q14‐15.^18^, ^19^ This antigen was identified by searching for surface molecules shared between trophoblast cells and tumor cells that they were hypothesized to be associated with fetus survival in the mother, or a tumor in the host as a semiallograft.^20^ Of particular note, 5T4 is expressed in high levels on the surface of trophoblast membranes, in addition to a variety of cancer cells and cancer stem cells (CSCs), but has a restricted pattern of expression in normal adult tissues.^21^ Clinical data showed that 5T4 is a marker of the poor prognosis in many cancers including ovarian cancer and is closely related to the progression and metastasis of tumors.^22^ The restricted profile of expression and biological function, undoubtedly, make 5T4 qualified as a promising target for immunotherapy in ovarian cancer.

In this study, we generated a second‐generation CAR‐T cell with the chimeric scFv derived from a high‐affinity 5T4 mAb named H6, which was screened by our research group through the hybridoma technique. 5T4 CAR‐T cells could elicit lytic cytotoxicity to target tumor cells in the 5T4‐dependent manner and secreted cytotoxic cytokines, including IFN‐γ, IL‐2, and GM‐CSF when compared with nontransduced (NT) T cells in vitro. Furthermore, adoptive transfer of 5T4 CAR‐T cells significantly delayed tumor formation in xenografts of peritoneal and subcutaneous animal models. One week after treatment, the tumor began to regress and did not recur for a period of time. These results provide evidence of the efficacy and feasibility of 5T4 CAR‐T cells and provide a theoretical basis for the clinical study of immunotherapy for ovarian cancer.

## 2 MATERIALS AND METHODS

### 2.1 Bioinformatic analysis

5T4 (*TPBG*) expression in normal ovary and ovarian cancer tissues at the mRNA level was compared using RNA‐seq data from Genotype‐Tissue Expression (GTEx) and the Cancer Genome Atlas (TCGA). RNA‐seq data from 88 normal ovaries and 419 primary ovarian cancer cases were collected. RNA‐seq data were represented as log2 (TPM+0.001), in which TPM refers to transcripts per million.

### 2.2 Cell lines

Ovarian cancer cell lines (ES2, SKOV3, OVCAR‐3, A2780, COC1, and HO8910), gastric carcinoma cell line AGS, colon cancer cell line LoVo, and lymphoma cell line Raji were purchased from the American Type Culture Collection (ATCC). All tumor cells were cultured in Roswell Park Memorial Institute (RPMI) 1640 medium supplemented with 10% fetal bovine serum (FBS) and 1% penicillin‐streptomycin, except in the case of ES2 and SKOV3 cells, which were cultured in Dulbecco's modified Eagle's medium (DMEM) high glucose medium containing 10% FBS. Human embryonic kidney (HEK) 293FT cells (obtained from Prof. Hanshuo Yang) were cultured in DMEM containing 10% FBS, 1% glutamax, and 1% penicillin‐streptomycin.

### 2.3 Construction of lentiviral expression vector targeting 5T4

The second generation of 5T4 CAR was constituted by the scFv fragment of antibody H6 (prepared by our group) as the extracellular antigen recognition region, in frame with a transmembrane domain and the 4‐1BB costimulatory and CD3ζ intracellular signaling domains. Then the target gene sequence was submitted to Shanghai ShengBo biopharmaceutical technology company for whole‐gene synthesis and introduced into vector PMT431 by homologous recombination to construct a lentiviral expression vector PSE3443 targeting the 5T4 antigen.

### 2.4 Lentivirus packaging, purification, and concentration

Opti‐MEM medium (Gibco, USA) was added to the packaging plasmid pCMV‐dR8.9 (65 µg), pCMV‐VSV‐G (35 µg), and the lentiviral expression vector PSE3443 (100 µg) containing the sequence of 5T4 CAR to prepare working liquids. HEK293T cells were cotransfected with this three‐plasmid system using Trans‐EZ transfection reagent (SunBio, Shanghai, China) to produce lentiviral supernatant. The lentiviral‐containing culture medium was collected in 36 and 48 h posttransfection by ultracentrifugation at 107 000×*g* for 2 h at 4°C. Quantitative PCR was used to detect virus titers using the DNA quantitative kit (Bio‐Rad, USA).

### 2.5 Generation and expansion of 5T4 CAR‐T cells

Human peripheral blood mononuclear cells (PBMCs) were isolated from peripheral blood of healthy volunteers by Ficoll‐Paque (GE Healthcare, USA) density gradient centrifugation. For the transduction of primary T cells, PBMCs were activated for 36 h by anti‐CD3/anti‐CD28 Dynabeads (Miltenyi Biotec, Germany) at a bead: cell ratio of 2:1 before infection. The activated T cells were transduced with lentiviral vectors at a multiplicity of infection of 10 in a 12‐well plate. The transduced T cells were cultured at a density of 1 × 10^6^ cells/mL in the presence of 100 IU/mL human recombinant IL‐2 (R&D, USA). Cell culture was monitored daily during transduction, and fresh media was added to maintain a cell concentration of (1‐2) × 10^6^ cells/mL. On day 3, following transduction, Dynabeads were removed.

### 2.6 Flow cytometry

We used a FACSCalibur instrument (NovoCyte, ACEA Biosciences, USA) and NovoExpress software (ACEA) for all flow‐cytometric analyses, analyzing at least 20 000 events and isotype antibodies were used as negative controls in all cases. Cells were gently washed twice with cold PBS prior to addition of antibodies. After 30 to 40 min of incubation at 4°C in the dark with fluorescent antibody, the cells were washed twice and resuspended with PBS prior to analysis. T cells phenotypes were analyzed with anti‐CD3 FITC, anti‐CD4 APC, and anti‐CD8 PE antibodies (BD Pharmingen, USA). The 5T4 antigen expression of tumor cells and transfection efficiency of CAR were detected using a FITC‐conjugated goat anti‐human antibody (ZSBIO, China).

### 2.7 Coculture of CAR‐T cells with ovarian cancer cells in vitro

5T4 CAR‐T cells were cocultured with different types of tumor cells (1 × 10^5^ cells/mL) at the effector‐to‐target (E/T) ratio of 1:1, 5:1, and 10:1 in a 96‐well plate for 24 h. NT T cells served as the control. After culture, the 96‐well plate was centrifuged at 1200×*g* room temperature for 5 min to isolate the cell supernatant. The specific in vitro antitumor activity of 5T4 CAR‐T cells was detected by the CytoTox96 nonradioactive cytotoxicity assay kit (Promega, USA). The remaining samples were analyzed for IFN‐γ, IL‐2, TNF‐α, IL‐6, and GM‐CSF secreted by CAR‐T cells using enzyme‐linked immunosorbent assay (ELISA) kits (BioLegend, USA) according to the manufacturer's instructions.

### 2.8 In vivo antitumor studies of 5T4 CAR‐T cells in peritoneal tumor model

All in vivo animal experiments were conducted and supervised by The Institutional Animal Care and Treatment Committee of State Key Laboratory of Biotherapy in Sichuan University. B‐NDG (NOD‐Prkdc^scid^ IL2rg^tm1^/Bcgen) severe immunodeficiency mice were purchased from the Beijing BIOCYTOGEN company at 5 to 6 weeks. First, after a week of adaptive breeding in our laboratory animal house, 200 µL SKOV3‐luc ovarian cancer cells (1 × 10^6^cells/mL) were intraperitoneal injected (i.p.) into B‐NDG mice to build a peritoneal tumor model on day 0. Two days after inoculation, tumor size was observed through bioluminescence imaging and mice were randomly divided into three groups of six. On day 2, mice were treated by intraperitoneal injection of 200 µL 5T4 CAR‐T cells or NT T cells (1 × 10^7^ cells/mL). The treatments were given every 2 days for a total of three times. In a follow‐up study, we built a second peritoneal model of ovarian cancer by injecting 200 µL OVCAR3‐luc cells (5 × 10^5^ cells/mL) into B‐NDG mice on day 0. On day 2, mice were treated with a single dose of 5T4 CAR‐T cells by i.p. at the E: T ratio of 2:1, 10:1, and 20:1, the minimum dose (2:1) was used for NT T cells. The blank control group was injected with an equal volume of saline. The weight of the mice was measured every 3 days. Bioluminescence imaging was performed weekly.

### 2.9 In vivo antitumor studies of 5T4 CAR‐T cells in subcutaneous tumor model

To build subcutaneous tumor transplantation model, 100 µL SKOV3‐luc ovarian cancer cells (5 × 10^6^cells/mL) were inoculated on the right upper limb back of mice on day 0. When the volume of tumor was about 20 mm^3^, mice were randomly divided into three groups of six. On day 5, mice were treated with 100 µL 5T4 CAR‐T cells or NT T cells (5 × 10^7^ cells/mL) injected through the tail vein. The treatments were given every 2 days for a total of three times. The blank control group was injected with an equal volume of saline. Tumor volume and mice weight were measured every 3 days (the formula *V* = *L* × *W* ^2^/2, where *L* and *W* represent the long and short diameters of the tumors, respectively). Bioluminescence imaging was performed weekly.

### 2.10 Statistical analysis

All results were reported as the mean ± standard deviation (SD) and analyzed by the unpaired *t*‐test. Data were plotted using GraphPad Prism software version 6.0c. *p*‐values < .05 were considered statistically significant.

## 3 RESULTS

### 3.1 5T4 oncofetal antigen expression was significantly upregulated in ovarian cancer compared to normal ovaries

**FIGURE 1:**
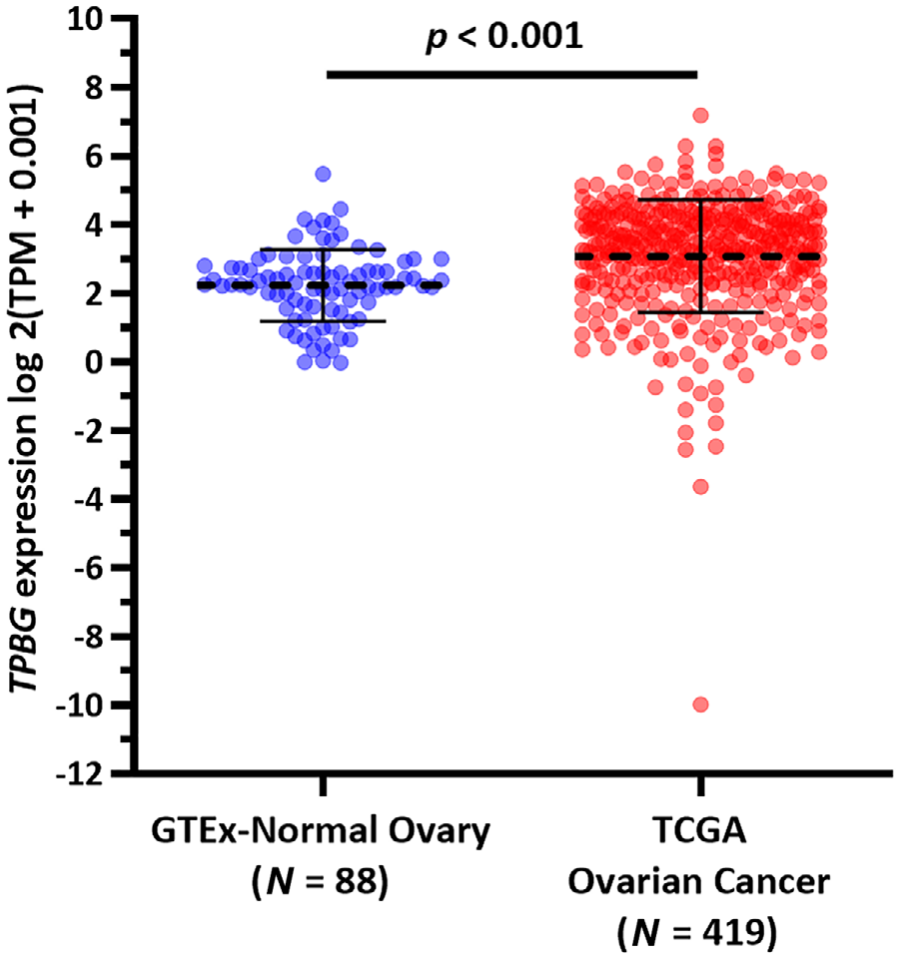
5T4 oncofetal antigen expression in normal ovary and ovarian cancer tissues at the mRNA level. 5T4 (*TPBG*) expression was significantly upregulated in the tumor tissues (p < .001)

By comparing RNA‐seq data of 88 normal ovaries in GTEx and 419 primary ovarian cancer cases in TCGA, we observed that *TPBG* expression was significantly upregulated in the tumor tissues (*p* < .001) (Figure 1). The median and 75% percentile of *TPBG* expression (log2(TPM+0.001)) in normal ovary group were 2.26 and 2.74, respectively. 326/419 (77.8%) and 278/419 (66.3%) of cancer cases had *TPBG* expression higher than 2.34 and 2.74, respectively.

### 3.2 Generation, expansion, and characterization of 5T4‐specific CAR‐T cells

**FIGURE 2:**
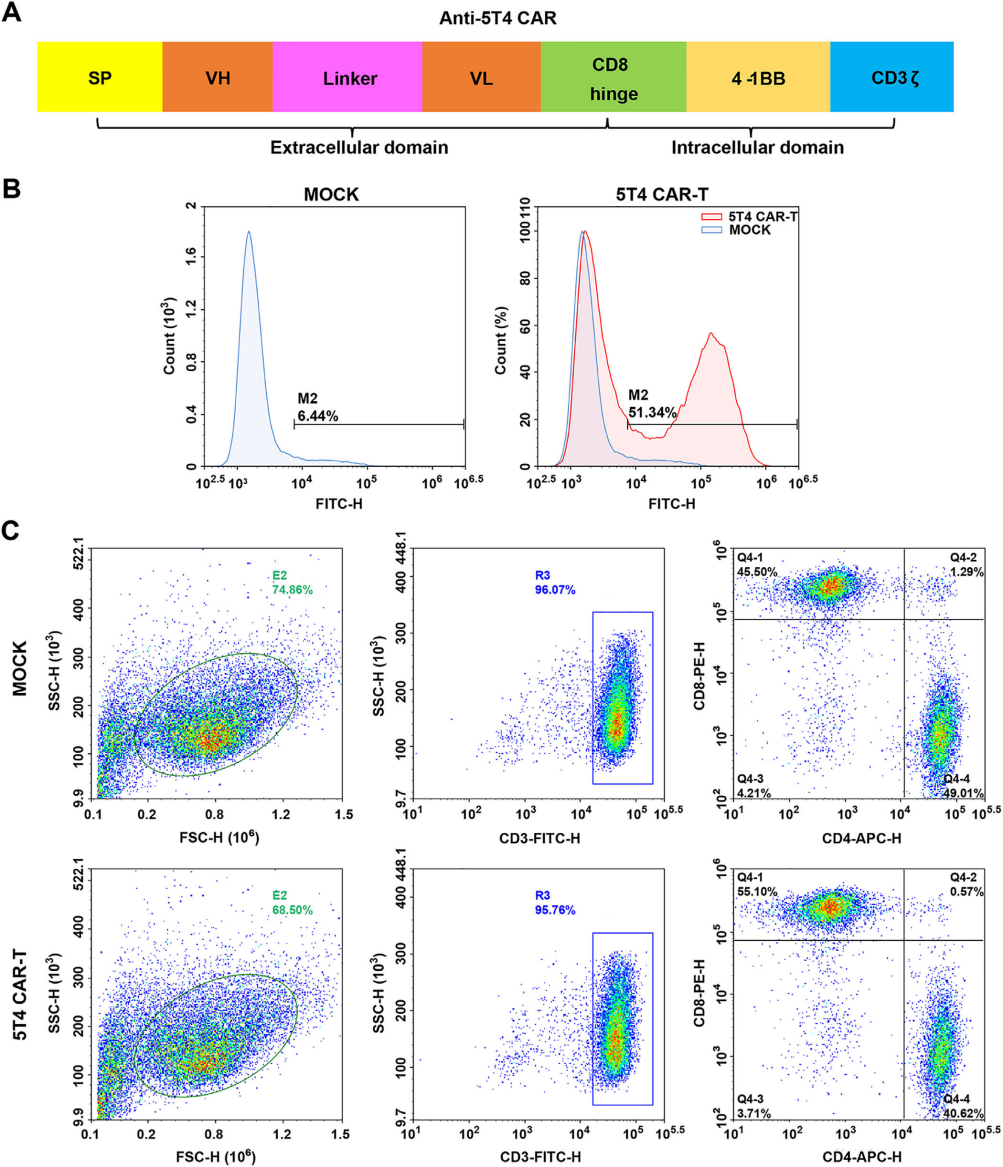
Generation, expansion, and characterization of 5T4‐specific CAR‐T cells. (A) Schematic representation of the second‐generation 5T4‐targeted CAR, which comprises the scFv derived from antibody H6 linked to a transmembrane domain fused to human costimulatory molecule 4‐1BB and CD3ζ intracellular signaling domains. (B) The transfection efficiency of 5T4‐specific CAR in T lymphocytes was detected by FCM analysis. (C) Phenotypic features of 5T4 CAR‐T and NT T cells (Mock group) were evaluated by FCM analysis

A second‐generation CAR consisting of a scFv fragment derived from antibody H6 and the signaling domains from costimulatory molecule 4‐1BB and the CD3ζ chain in sequence (Figure 2A) was inserted into a lentiviral vector system. Constructed lentiviruses were cotransfected into HEK293T package cells with a three‐virus system. The average virus titer is 7.85 × 10^8^ TU/mL. PBMCs were isolated on day 0 and lentivirus‐mediated CAR gene transfection was performed on day 2. The transfection efficiency of 5T4 CAR‐T cells was approximately 52% (Figure 2B) verified by flow cytometric (FCM) analysis on day 5. There was a large population of CD3^+^ T cells (more than 95%) in both NT T and CAR‐T cell groups. While the proportion of CD3^+^CD4^+^ T cells in NT T cells and CAR‐T cells is was 49% and 41%, respectively. The proportion of CD3^+^CD8^+^ T cells in NT T cells and CAR‐T cells is 45% and 55%, respectively (Figure 2C). In the CAR‐T cells group, CD8^+^ T cells were dominant relative to CD4^+^ T cells.

### 3.3 Cytotoxicity of 5T4 CAR‐T cells on 5T4^+^ cancer cell lines in vitro

**FIGURE 3:**
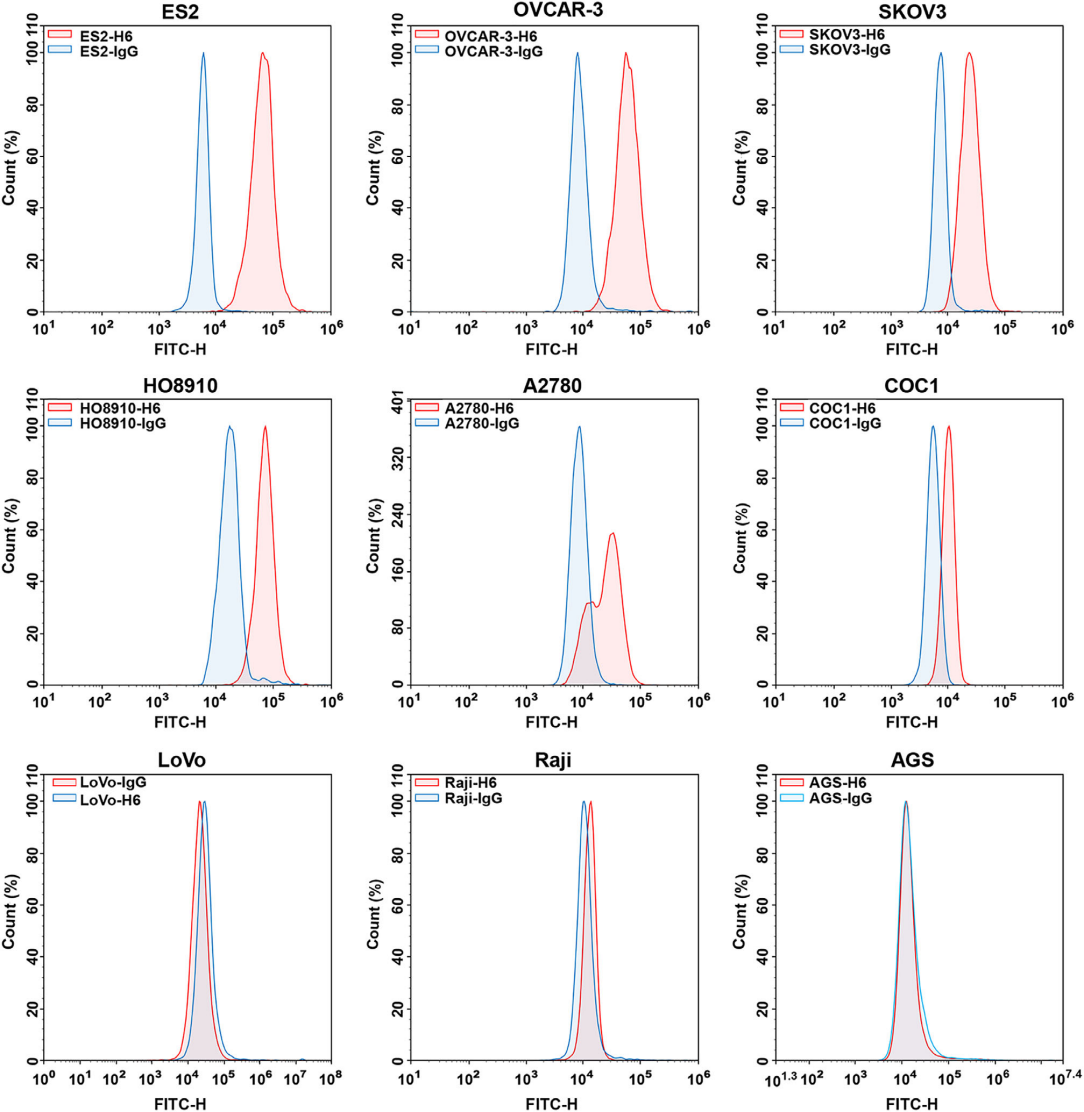
5T4 protein expression on the surface of ovarian cancer cells. FCM analysis of tumor cells with isotype IgG (blue) or H6 antibody (red), respectively. Since all ovarian cancer cell lines were positive for 5T4 expression, three additional negative cell lines were added

**FIGURE 4:**
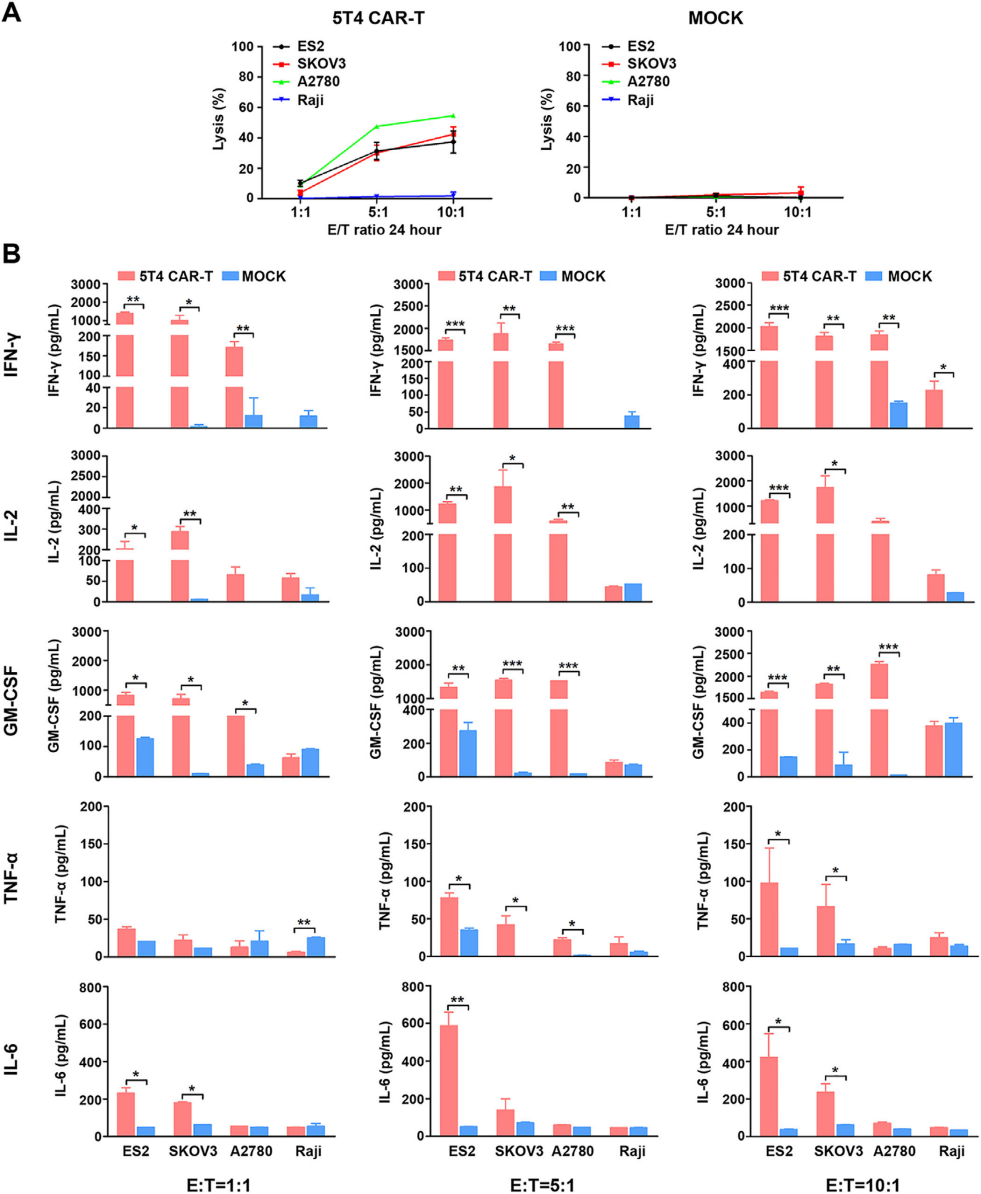
In vitro cytolytic activities of 5T4‐targeted CAR T cells. The cytotoxicity of T cells expressing 5T4‐specific CAR was determined by coculture with three 5T4^+^ ovarian cancer cell lines and one 5T4^−^ cell line at varying E/T ratios (1:1, 5:1, and 10:1) for 24 h, respectively. (A) Cell lysis was determined by a standard nonradioactive cytotoxic assay. 5T4 CAR‐T cells can specifically kill 5T4 positive tumor cells but not the negative cells. Each data reflect the mean ± SD of triplicates. (B) The cytokine concentration in the media was determined by ELISA. 5T4 CAR‐T cells produced IL‐2, IFN‐γ, and GM‐CSF after stimulation with 5T4‐positive tumor cells. No cytokine release was seen with NT T cells

The surface expression of 5T4 antigen was assessed by FCM analysis in a series of cancer cell lines. All ovarian cancer cell lines were 5T4‐positive. Among them, 5T4 was highly expressed on the surface of ES2, SKOV3, OVCAR‐3, HO8910, moderately expressed in A2780, and poorly expressed in COC1. AGS, LoVo, and Raji cell lines were 5T4 negative (Figure 3). CAR‐T cells were cocultured with target tumor cells at E/T ratios of 1:1, 5:1, and 10:1 for 24 h. The lactate dehydrogenase release assay was used to evaluate the cytotoxicity of 5T4 CAR‐T cells against 5T4^+^ ovarian cancer cells. 5T4 CAR‐T cells exhibited specific killing activity against 5T4^+^ ES2 cells (34.89%), SKOV3 cells (48.03%), and A2780 cells (54.12%) at the E/T ratio of 10:1, while no effect on 5T4^−^ Raji cells was observed (Figure 4A). Moreover, the cytotoxicity of 5T4 CAR‐T cells was increased as the E/T ratio increased. The cytotoxicity of CAR‐T cells depends, but not linearly dependent, on the expression level of 5T4 on the tumor cell surface. This may be related to the state of tumor cells and their sensitivity to CAR‐T cells.

### 3.4 Cytokine secretion of 5T4 CAR‐T cells

The remaining cell supernatant was isolated after centrifugation and was used to detect the level of cytokines released by CAR‐T cells, including IFN‐γ, IL‐2, GM‐CSF, TNF‐α, and IL‐6. Compared to NT T cells, 5T4 CAR‐T cells produced significantly higher levels of IL‐2 (1500‐2000 pg/mL), IFN‐γ (about 2000 pg/mL) and GM‐CSF (1600‐2300 pg/mL), a little bit higher levels of TNF‐α (about 100 pg/mL) and IL‐6 (40‐300 pg/mL) when cocultured with 5T4^+^ ovarian cancer cells at the E/T ratio of 10:1 (Figure 4B). There are many factors that influence the type and quantity of cytokines secreted by CAR‐T cells, in particular, the structural design of CARs and the T cell subtypes. Prior studies have shown that different costimulatory molecules may result in different cytokine profiles, for example, introduction of CD28 led to higher degree Th1 cytokines (IL‐2, IFN‐γ, TNF‐α, and GM‐CSF) than 4‐1BB, and CD8^+^ T cells prefer to produce IFN‐γ while CD4^+^ T cells prefer to produce IL‐2 and TNF‐α.^23^

### 3.5 Antitumor activity of 5T4 CAR‐T cells in peritoneal tumor models

**FIGURE 5:**
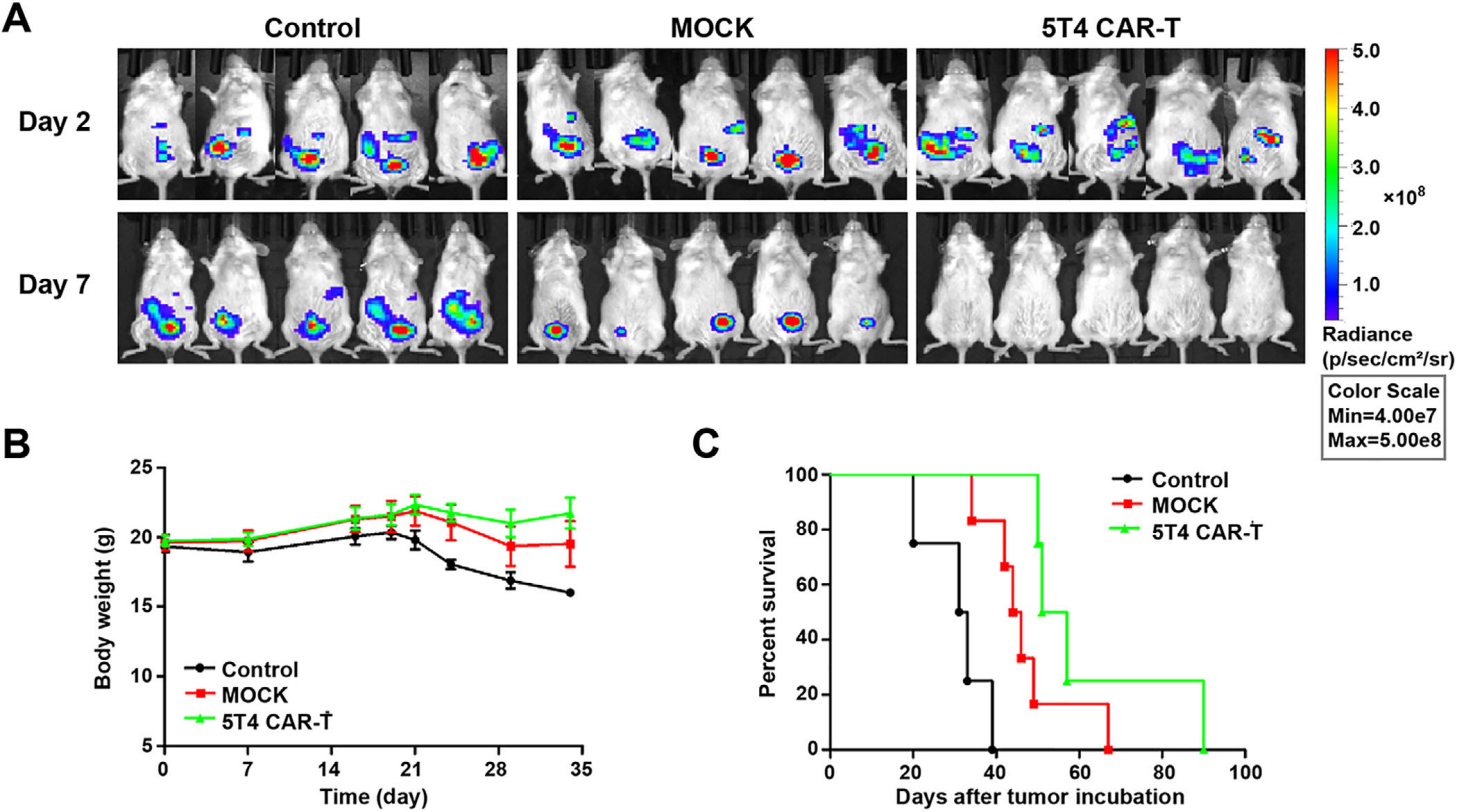
Antitumor activity of 5T4 CAR‐T cells in peritoneal tumor model. (A) 2 × 10^5^ SKOV3‐luc cells were intraperitoneally injected into B‐NDG mice on day 0, and 2 × 10^6^ 5T4 CAR‐T cells were intraperitoneally injected 2 days later for treatment. The treatments were given every 2 days for a total of three times. We observed the growth of peritoneal tumors by bioluminescence imaging weekly. (B) Body weight curve of mice. (C) The overall survival of mice treated with the 5T4 CAR‐T cells, NT T cells or saline

**FIGURE 6:**
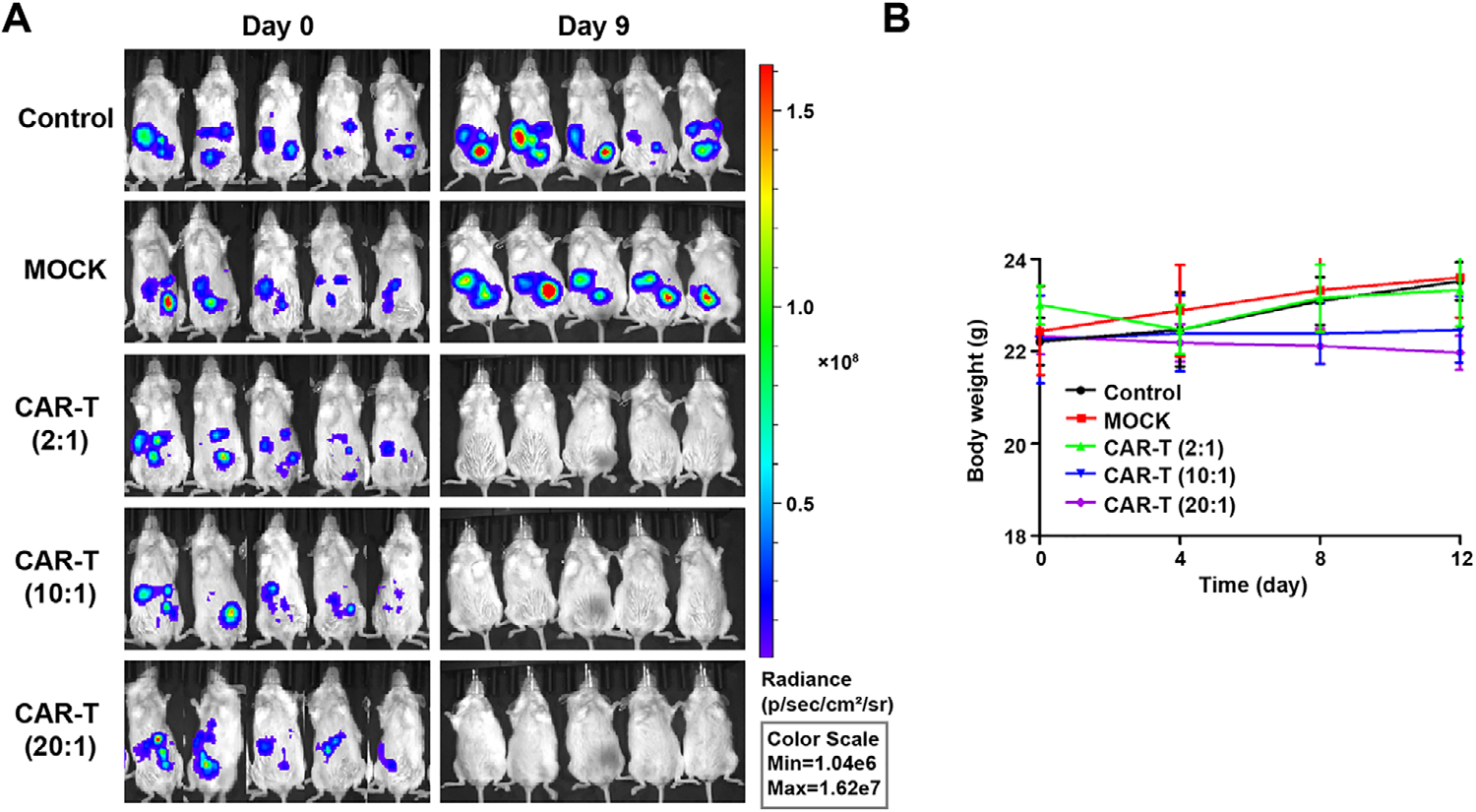
Antitumor activity of 5T4 CAR‐T cells in a second peritoneal tumor model. (A) 1 × 10^5^ OVCAR3‐luc cells were intraperitoneally injected into B‐NDG mice on day 0. On day 2, mice were treated with a single dose of 5T4 CAR‐T cells by i.p. at the E/T ratio of 2:1, 10:1 and 20:1, the minimum dose (2:1) was used for NT T cells. (B) Body weight curve of mice

After verifying the significant cytotoxicity of 5T4 CAR‐T cells in vitro, we further investigated whether these CAR‐T cells could eradicate tumor cells in vivo. B‐NGD mice were challenged via the intraperitoneal route with 2 × 10^5^ SKOV3‐luc cells on day 0 and treated with 2 × 10^6^ CAR‐T cells on day 2 when tumor formation was first observed by bioluminescence imaging. In the group treated with saline, the tumor continued to proliferate (Figure 5A), mice gradually lost weight (Figure 5B), which resulted in a short survival time. The first death occurred on day 20, and the mice all died within 39 days (Figure 5C). In contrast, in the group treated with 5T4 CAR‐T cells, according to the bioluminescence imaging, the tumor subsided rapidly within 1 week while tumors regressed slowly and recurred after 1 month in the group treated with NT T cells. These results indicate that 5T4 CAR‐T cells therapy has effective antitumor activity in vivo and can eradicate peritoneal tumors completely in a short time without recurrence, which may be related to the production of memory cells. This conclusion was also verified in another peritoneal model of ovarian cancer (OVCAR3), demonstrating that 5T4 CAR‐T cells can cause tumor regression at very low doses (E/T = 2:1) while tumors in the control groups continued to grow (Figure 6).

### 3.6 Antitumor activity of 5T4 CAR‐T cells in subcutaneous tumor models

**FIGURE 7:**
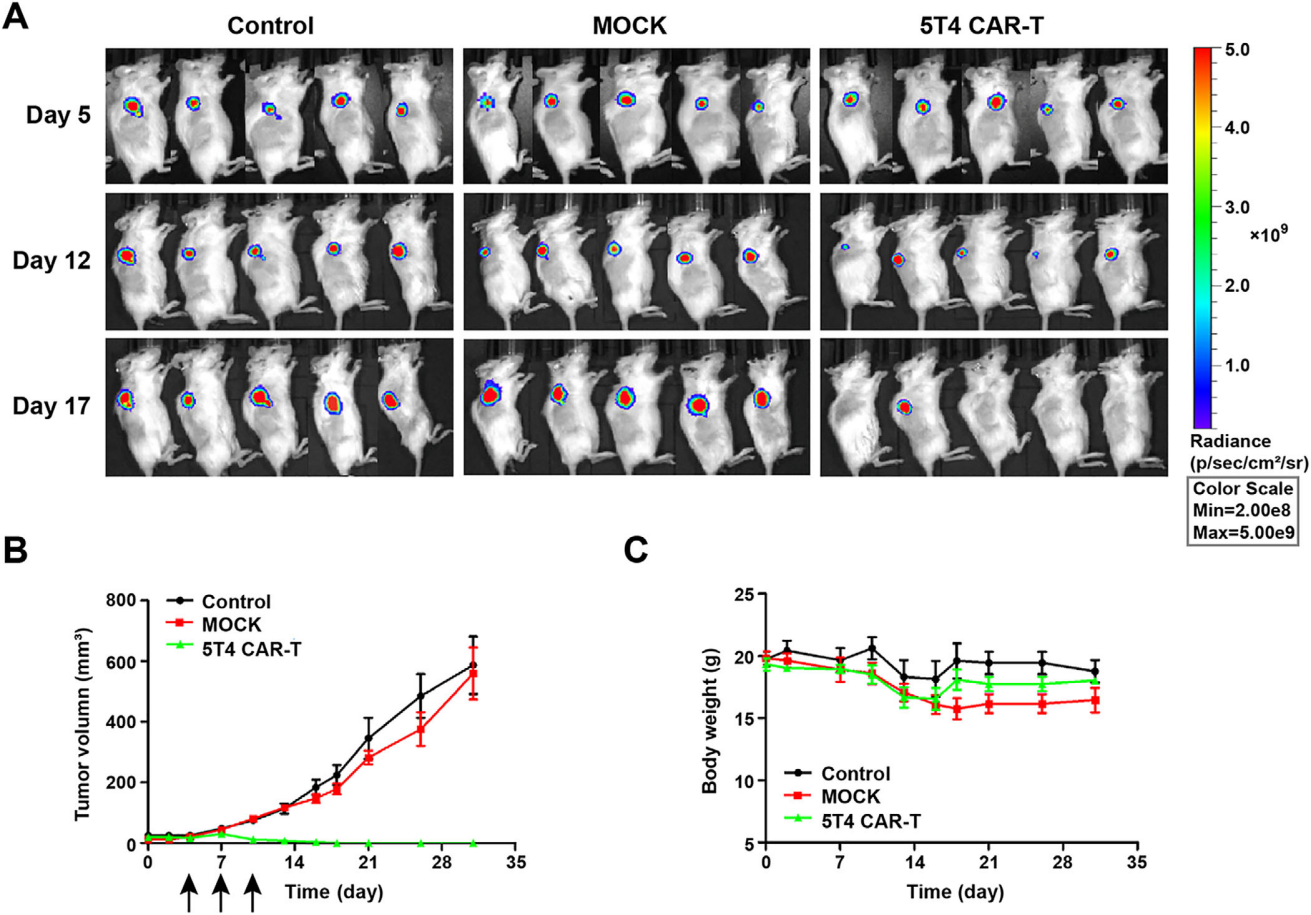
Antitumor activity of 5T4 CAR‐T cells in subcutaneous tumor model. (A) On day 0, 5 × 10^5^ SKOV3‐luc cells were injected subcutaneously into the right back of B‐NDG mice. When the tumor volume was about 20 mm^3^, mice were treated with 5 × 10^6^ CAR‐T cells. The treatments were given every 2 days for a total of three times. We observed the growth of subcutaneous tumors by bioluminescence imaging weekly. (B) Tumor volume growth curve. We measured tumor volume with a Vernier caliper (the formula *V* = *L* × *W* ^2^/2, where *L* and *W*, respectively, represent the long and short diameters of the tumors) every 3 days. (C) Body weight curve of mice

Ovarian cancer cells were implanted subcutaneously in B‐NDG mice in order to test the antitumor activity of 5T4 CAR‐T cells in vivo. 5 × 10^5^ SKOV3‐luc cells were injected subcutaneously into the right back of mice on day 0. When the tumor volume reached about 20 mm^3^, mice were treated with 5 × 10^6^ CAR‐T cells via the tail vein. In groups treated with saline or NT T cells, the tumor volume increased gradually and mice had a short survival time. The first deaths occurred on day 22 and day 33, respectively. Mice in both groups all died within 44 days. For the 5T4 CAR‐T cells group, however, tumor volume decreased significantly 1 week after treatment, and completely subsided within 2 weeks (Figure 7A and 7B). In subcutaneous tumor model, 5T4 CAR‐T cells also delayed tumor formation in mice, whereas the effect was inferior to that in peritoneal tumor model and the NT T cells had no effect on tumor growth. Perhaps because T cells need to identify and infiltrate tumor through systematic circulation after intravenous injection, the number of effector T cells reaching the tumor site is greatly reduced. It may be that the immunosuppressive microenvironment around solid tumor promotes exhaustion of T cells, which increases the difficulty of cell therapy.

## 4 DISCUSSION

As one of the most attractive immunotherapy strategies in recent years, CAR‐T cell therapy has shown great benefits in patients suffering from B cell malignancies. The HLA‐independent antigen recognition region and intracellular T cell activation signal modules were combined as fusion receptors to construct genetically engineering T cells against malignant cells. The extracellular antigen‐binding domain is usually derived from the variable region of a mAb targeting tumor‐related antigen. Therefore, searching for tumor‐specific target antigens is a critical first step in the design and construction of CARs.

5T4 seems to be an ideal therapeutic target for its selective expression on the surface of cancer cells and CSCs, its association with a tumor‐initiating phenotype, and involvement in tumor progression. CSCs are a subpopulation of tumor cells that have the ability to maintain vitality through self‐renewal and infinite proliferation, which are thought to be involved in the process of tumor germination, progression, further metastasis, and even recurrence.^24^ 5T4‐targeted vaccines and antibody‐drug conjugates can kill CSCs and non‐CSCs effectively and have shown good preclinical and clinical effects without serious toxicity.

Previously, we have screened out a high‐affinity antibody against 5T4 and evaluated efficacy of the antibody‐drug conjugate H6DM4 in gastrointestinal tumor xenograft models.^25^ The affinity of antibody H6 was 1.871 × 10^−11^ mol/L measured by Biacore analysis, which was two orders of magnitude higher than other antibodies. It is reported that the higher affinity results in greater levels of cytokine secretion and a better therapeutic effect of CAR‐T cells in vivo.^26^ Additionally, higher affinity CARs can recognize the low‐density antigen on the tumor surface and reduce the occurrence of tumor immune escape.

Poor T cell persistence is one of the many limitations of clinical CAR‐T cell therapy. The addition of costimulatory receptors greatly improved cytotoxicity and survival in the second generation of CAR T cells. Interestingly, the choice of different costimulatory molecules has different effects on the T cell function. Published clinical studies have shown that CAR‐T cells containing CD28 persisted for 1–3 months in vivo, while CAR‐T cells containing 4‐1BB persisted for 5 years in vivo.^27^ This suggests that 4‐1BB may be a superior option for preparing CAR‐T cells because it can maintain T cell activation and survival.

Therefore, here we extend our work to ovarian cancer, incorporating 4‐1BB costimulation signaling domain into CAR construct based on chimeric antibody H6 scFv, which retains the affinity and specificity of mouse antibody while the introduction of a constant region of human antibody reduced the immunogenicity. Safety has always been one of the most important issues in CAR‐T research. Whether H6 scFv has some degree of toxicity and immunogenicity needs to be further demonstrated in subsequent clinical trials.

In this study, our cell coculture experiment confirmed that 5T4 CAR T cells have a specific concentration‐dependent killing effect on 5T4^+^ ovarian cancer cells in vitro and can secrete Th1 cytokines (IL‐2, IFN‐γ) response upon specific antigen encounter. IL‐2 can promote the activation of T cells and enhance the killing effect of cellular immunity, which is very critical for CAR‐T cell proliferate and differentiate into effector T cells. IL‐2 not only promotes the maturation and differentiation of T cells, but also other types of cells, such as natural killer (NK) cell. Activated CAR‐T cells secrete IFN‐γ, an essential factor that regulates a variety of aspects of immune system responses, can play a direct or indirect role in inducing apoptosis of tumor cells.

In the mice of peritoneal xenotransplanted tumor models, we observed the clinical characteristics of ovarian cancer, spreading to peritoneal cavity and forming bloody ascites. We verified that 5T4 CAR‐T cells had a notable therapeutic effect in this model as tumor abrogation in the intraperitoneal metastatic ovarian cancer model emerged immediately just 1 week after CAR‐T cells injection, extending mouse survival. Regional i.p. delivery shows better curative effect due to it subtly avoids transport restrictions, effectively redistributing 5T4 CAR‐T cells to tumor sites, potentially reducing their systemic distribution, and avoiding systematic targeting of T cells in normal tissue, further reducing the risk of unnecessary toxicity.^28^, ^29^

Moreover, we also found that the systematic delivery of 5T4 CAR‐T cells in the subcutaneous injection xenotransplanted model significantly controlled the growth of Skov3 ovarian cancer. Tumor regression quickly appeared 2 weeks after CAR‐T cells delivery by tail vein injection. The same pattern was not observed in the control group, where tumors grew in size. This model confirmed that T cell persistence was attainable via systemic T cell delivery, suggesting the capacity of 5T4 CAR‐T cells to circulate, focus on tumors, expand, and perform antitumor functions.

In conclusion, our study demonstrates that 5T4 is another valid target in ovarian cancer besides to Her2. The functional characterization of our 4‐1BB costimulated 5T4 CAR provides a basis for clinical studies on adoptive CAR‐T cell therapy for a wide range of 5T4 overexpressed tumors. Furthermore, we have shown that administration of CAR‐T by regional delivery can accelerate tumor regression. However, in this study, the overall survival time of the mice was not significantly prolonged in SKOV3‐luc models, and the mice in the treatment group began to die later. Thus, we constructed an additional peritoneal model using OVCAR3‐luc cells, and demonstrated the effectiveness of 5T4 CAR‐T cells which can cause tumor regression at minimum dose (E/T = 2:1), suggesting that we may be able to use lower doses for better safety. Clinical side effects of CAR‐T therapy have been of concern, including cytokine releasing storms, neurotoxicity, and off‐target effects. Further work we aim to assess the safety of 5T4 CAR‐T cells, through adjust the structure of CARs, in combination with immunosuppressive drugs such as glucocorticoids, or though the reduction in the amount and frequency of CAR‐T cell doses to reduce toxicity.

## CONFLICT OF INTEREST

The authors declare no conflict of interest.

## AUTHOR CONTRIBUTIONS

**Conception and design**: Jinliang Yang, Hanshuo Yang, Cuiyu Guo, E Dong.

**Development of methodology**: Cuiyu Guo, E Dong, Qinhuai Lai, Shijie Zhou, Guangbing Zhang, Mengdan Wu, Xiaozhu Yue.

**Data acquisition**: Cuiyu Guo, Mengdan Wu, Yiran Tao, Yujia Peng, Ying Lu, Yuyin Fu, Weirong Lai, Zhixiong Zhang.

**Analysis and interpretation of data**: Cuiyu Guo, E Dong, Guangbing Zhang, Xiaozhu Yue, Yiran Tao, Yujia Peng, Ying Lu, Yuyin Fu.

**Writing, review, and/or revision of the manuscript**: Cuiyu Guo, E Dong, Qinhuai Lai, Shijie Zhou, Guangbing Zhang, Jamel Ali, Jinliang Yang, Hanshuo Yang.

**Study supervision**: Jinliang Yang, Hanshuo Yang, Yuqin Yao, Fanxin Ma, Lantu Gou.

5

## Data Availability

Supporting data of this study are available from the corresponding author on reasonable request. Source data for graphs and tables presented in this manuscript are provided as a Source Data file.
